# Genetic Structure, Linkage Disequilibrium and Signature of Selection in Sorghum: Lessons from Physically Anchored DArT Markers

**DOI:** 10.1371/journal.pone.0033470

**Published:** 2012-03-13

**Authors:** Sophie Bouchet, David Pot, Monique Deu, Jean-François Rami, Claire Billot, Xavier Perrier, Ronan Rivallan, Laëtitia Gardes, Ling Xia, Peter Wenzl, Andrzej Kilian, Jean-Christophe Glaszmann

**Affiliations:** 1 UMR AGAP, CIRAD, Montpellier, France; 2 Diversity Arrays Technology Pty Ltd., Yarralumla, Australia; University of Guelph, Canada

## Abstract

Population structure, extent of linkage disequilibrium (LD) as well as signatures of selection were investigated in sorghum using a core sample representative of worldwide diversity. A total of 177 accessions were genotyped with 1122 informative physically anchored DArT markers. The properties of DArTs to describe sorghum genetic structure were compared to those of SSRs and of previously published RFLP markers. Model-based (STRUCTURE software) and Neighbor-Joining diversity analyses led to the identification of 6 groups and confirmed previous evolutionary hypotheses. Results were globally consistent between the different marker systems. However, DArTs appeared more robust in terms of data resolution and bayesian group assignment. Whole genome linkage disequilibrium as measured by mean r^2^ decreased from 0.18 (between 0 to 10 kb) to 0.03 (between 100 kb to 1 Mb), stabilizing at 0.03 after 1 Mb. Effects on LD estimations of sample size and genetic structure were tested using i. random sampling, ii. the Maximum Length SubTree algorithm (MLST), and iii. structure groups. Optimizing population composition by the MLST reduced the biases in small samples and seemed to be an efficient way of selecting samples to make the best use of LD as a genome mapping approach in structured populations. These results also suggested that more than 100,000 markers may be required to perform genome-wide association studies in collections covering worldwide sorghum diversity. Analysis of DArT markers differentiation between the identified genetic groups pointed out outlier loci potentially linked to genes controlling traits of interest, including disease resistance genes for which evidence of selection had already been reported. In addition, evidence of selection near a homologous locus of FAR1 concurred with sorghum phenotypic diversity for sensitivity to photoperiod.

## Introduction

Identifying the genetic factors controlling variability in traits of agronomic and adaptive importance constitutes the basis for sustainable management of genetic resources. Such management is of central importance, not only in a short-term breeding perspective, but also to guarantee conservation of the genetic diversity currently available. Association mapping, also known as linkage disequilibrium mapping, is an efficient strategy for dissecting the genetic control of phenotypic variability down to the gene scale (for reviews see [1], [2]). It exploits large allelic diversity and historical recombination events, providing increased mapping resolution of regions expected to be associated with the traits of interest in a wide range of genetic backgrounds. As a prerequisite, geneticists need to clearly understand the history of successive bottlenecks, migrations, adaptations and human selection events that shaped their association panels. Admixed materials that diverged since domestication and are pooled together in the same statistical analysis can lead to spurious associations when the trait is differentiated between genetic subgroups. Once the genetic structure has been clearly understood, the ability to identify genes underlying the genetic variability of the target trait depends on the degree of Linkage Disequilibrium (LD) in the studied population and on the marker density available. In this context, accurate estimates of the LD breakdown window need to be acquired.

Alongside the exploitation of LD as a genome mapping approach, the detection of outlier loci concerning genetic differentiation provides a complementary strategy for identifying genome regions and candidate genes related to adaptive traits. The challenge consists in distinguishing loci harbouring patterns of differentiation that are significantly different from the one expected under realistic neutral demographic scenarios.

Sorghum is one of the world's most important cereals for human and animal nutrition. It currently ranks fifth for grain production tonnage. In developing countries, it is a major staple food and fodder crop constituting one of the pillars of food security. In developed regions, it has been primarily grown for animal feed. In addition, its merits as a bioenergy crop have recently been highlighted [3], [4]. Its success is mainly due to its high level of drought tolerance and its adaptation to a large array of environmental conditions and uses. The recent release of its genome sequence [5], its phylogenetic proximity with several important C4 species (maize, switchgrass, sugarcane) and its low genome complexity, contribute to its interest on a more fundamental level. In this context, it is important to develop a clear framework to identify the genes of economic and adaptive interest in sorghum, through association mapping or selection scanning.

Characterization of worldwide sorghum molecular diversity has mainly been based on the analysis of two large and representative panels. Deu *et al.* [6] and Caniato *et al.* [7] analysed a worldwide core collection of 210 accessions established to take race classification, latitude of origin, response to day length and crop management into account. Casa *et al.* [8] and Brown *et al.* [9] analysed a panel of 216–228 converted lines corresponding to exotic lines that have been introgressed with photoperiod-insensitivity and dwarfing alleles [10]. These analyses were done with relatively limited numbers of multi-allelic markers (60 RFLP probes in [6], 38 SSR in [7], 47 SSR in [8]), recently complemented with larger numbers of bi-allelic (SNP) markers in the study by Brown *et al.* [9] (303 SNP and 38 SSR). These different studies converged in identifying genetic groups that correspond to racial and geographical origins. However, slight differences were obtained on a fine scale, and the stability of the genetic structure estimations provided by different marker types and numbers has not been thoroughly assessed.

The level and extent of LD has yet to be accurately determined in sorghum. So far, in addition to the pioneer study by Hamblin *et al.* [11], which explored LD evolution within short sequences of 400 bp (95 regions sequenced), the most relevant analysis has been based on a reduced set of single nucleotide polymorphisms (249 SNP) on six small genomic regions ranging from 38 to 102 kb among 32 cultivated and wild accessions [12]. This study revealed that LD could expand to medium range (up to 100 kb), but generally markedly decayed within a distance of 15 kb.

The search for genomic regions and genes affected by natural and artificial selection has been launched in a series of studies [11], [13]–[15] based on more than 300 genomic regions corresponding either to anonymous genetically mapped loci (95 in [11], 204 in [13]), or to genes of the starch and kafirin metabolism pathways [14], [15] in small panels of accessions including fewer than 40 cultivated accessions. Despite the recent domestication and selection history of sorghum, these studies did not identify any clear evidence of selection in the studied loci, possibly due to the lacks of statistical power (low number of polymorphisms per fragment and small panel of accessions analysed) and of an appropriate neutral model. Casa *et al.* [16] analysed the diversity of 98 microsatellite markers in a larger panel including 73 landraces and 31 wild and weedy accessions and, using different statistical methods, they concluded that 15% of the markers analysed harboured evidence of selection. Finally, de Alencar Figueiredo *et al.* [17] analysed the diversity of 6 genes potentially involved in grain quality within a subsample of 53 cultivated accessions of the worldwide collection already described by Deu *et al.* [6] and highlighted the detection of a signature of molecular selection at 3 loci using Tajima's D test. To date, whole-genome scans have yet to be reported in sorghum.

The recent development of sorghum DArT markers [18]–[20] provides a new opportunity to refine current perceptions and develop approaches towards whole-genome scale analyses. The goals of this study were to i) physically map DArT markers on the sorghum reference sequence, ii) compare the properties of DArT markers with SSRs and RFLPs in relation to the description of the diversity of a worldwide sorghum collection, iii) provide estimates of the extent of LD on a whole-genome scale on the same collection, iv) assess the sensitivity of LD patterns to sample composition, and v) assess the potential of the current DArT coverage to detect genomic regions potentially subjected to selection events.

## Methods

### Plant material

We used the panel developed by Deu *et al* [6] which is representative of the diversity of sorghum landraces (Table S1). The establishment of this core sample (CS) was based on race classification, latitude of the country of origin, response to day length and production systems. This panel includes representatives of the five basic races of cultivated sorghum (B: bicolor, C: caudatum, D: durra, G: guinea and K: kafir) and intermediates between them.

### RFLP and SSR genotyping

The RFLP data presented in this study correspond to a subset (60 probes, 172 RFLP bands) of those produced by Deu *et al.* [6] described in de Alencar Figueiredo *et al.* [21]. In addition, the CS was genotyped with forty SSRs evenly spread throughout the genome developed under the Generation Challenge Programme (Billot et al. submitted). Compared to the list available at [http://sat.cirad.fr/sat/sorghum_SSR_kit/], 8 SSR (gpsb069, gpsb089, gpsb148, gpsb151, Xcup62, Xtxp012, Xtxp295 and Xtxp339) were not used. Protocols are available at the same address.

### DArT genotyping

We used a *Pst*I-*Ban*II DArT genotyping array built from 92 accessions including worldwide breeding lines, landraces, and wild/weedy sorghums [19], [20]. We extended it in this study with 71 additional accessions (Table S1).

In addition, a second genotyping array was developed from a Mite/*Bsp*1286I complexity reduction based on a mixture of 87 accessions (see Table S1).

One hundred and ninety genotypes from the CS were analysed in two sets of 95 accessions, both representative of the 10 clusters described by Deu *et al* (2006) [6]. For each accession, genotyping with the *Pst*I/*Ban*II array was performed as described in Mace *et al* [20]. Genotyping with the Mite/*Bsp*1286I array was performed as follows. Genomic DNA was digested with *Bsp*1286I. *Bsp*1286I adapters were ligated to the digested DNA with T4 DNA ligase (NEB). A 1-µl aliquot of the ligation product was used as the template in two amplification reactions with one DArT-*Bsp*1286I primer (5′-GAG TAG TGC CAG AAC GGT C-3′) and two MITE (transposable elements) [22] DArT-TIR specific primers (5′-TTT TTG GAA CTA AAC AAG GCC-3′ and 5′-G GGT GAA CTA AAC AAG GCC-3′). In a first PCR, for one unit of *Bsp*1286I primer, we used 10 units of TIR primers to maximize the number of different TIR fragments produced. In a second PCR, for 10 units of *Bsp*1286I primer, we used 10 units of TIR primers to amplify TIR-*Bsp*1286I fragments.

The hybridization mixtures were denatured and hybridized to the two different DArT microarrays described above, which contained 6244 clones each. Two controls were used for the *Pst*I-*Ban*II chip, 8 for the *Bsp*1286I chip. DArT markers were scored according to standard DArTsoft protocols.

### Anchoring DArT markers to the sorghum genome sequence

A total of 2413 polymorphic DArT markers identified in different diversity and mapping analyses were sequenced and assembled with TGI Clustering tools (TGICL) [23] in order to eliminate redundancy due to random production of clones. GenBank accession numbers (FI847678 through FI849555) corresponding to the different clones are referenced in Table S2. Non-redundant DArT marker sequences were aligned on sorghum genome pseudomolecules (ftp://ftp.jgi-psf.org/pub/JGI_data/Sorghum_bicolor/v1.0/Sbi/). Sequences displaying less than 80% similarity with the sorghum genome or corresponding to highly repeated regions were eliminated. Information regarding clone redundancy, contig sequences, Blast quality results, position of the markers on the sorghum genome (physical and genetic maps) are available in Table S2.

### Diversity and Structure analyses

According to the low residual heterozygosity observed for SSR (2.7%), which is consistent with the several generations of selfing applied to the analysed accessions, all the analyses were performed considering the accessions as homozygotes. For heterozygous accessions at RFLP and SSR markers, one of the two alleles was randomly sampled.

The genetic structure of the analysed collection was estimated using the model-based Bayesian clustering method implemented in STRUCTURE software version 2.1 [24]. Allele frequencies in each of the K clusters (from 2 to 15) were estimated, and for each accession, the percentage of its genome derived from each cluster was estimated. We assumed a single domestication event and restricted our analysis to the correlated frequency model [25]. We set other parameters at their default values using the admixture model and infer alpha option. We used a 3.10^4^ burn-in period and 10^5^ iterations for DArTs, and a 10^6^ burn-in period and 10^6^ iterations for RFLPs and SSRs, as these parameters resulted in relative stability of the results with 10 runs per K value. The genome composition (genome plot) of each accession was represented for each K value and each marker system. Only accessions displaying a membership larger than 0.6 were assigned to a genetic group, resulting in the assignment of 80% of the accessions. In order to compare the stability of accession assignments to the different genetic groups, either between runs of the same marker system or between marker systems for a given structure level (K), the dissimilarity index of ancestry per individual was computed according to the formula [26]:
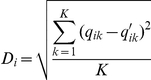
where q_ik_ and q′_ik_ correspond to the assignment proportion of accession i to group k according to two different runs or marker systems.

**Figure 1:**
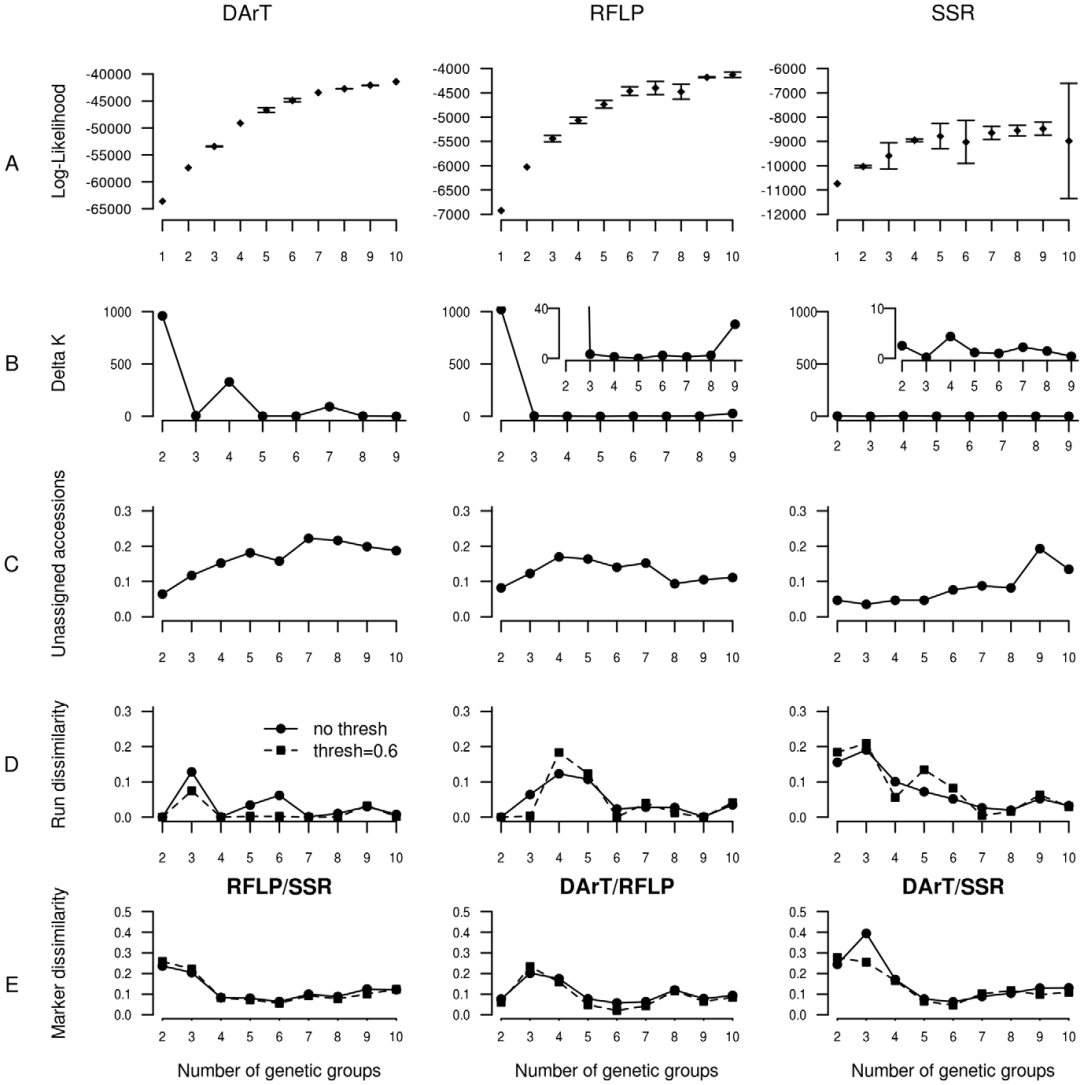
Analysis of sorghum CS using DArTs, RFLPs and SSRs. (A) Average log-likelihood and standard errors obtained with STRUCTURE software. For the three marker systems, the log-likelihood reached a plateau around K = 6. (B) The ΔK parameter [69] enabled the identification of high values at K = 2 (DArTs, RFLPs) and K = 4 (DArTs, SSRs), and lower peaks at K = 7 (DArTs, SSRs) and K = 6 (RFLPs, although this peak was not visible with the Y axis-scale used). (C) Proportion of unassigned accessions at the 0.6 membership threshold for each marker system. This proportion varied with the marker systems and the number of groups, K. To assess the stability of accession assignments to the different genetic groups between runs for the different marker systems, the average dissimilarity indices between runs for all accessions were computed and are reported in (D) according to calculations presented in the Material and Methods section. This analysis revealed that SSR markers provided the least stable assignment across runs for the same K level. Lastly, assignment stability across marker systems is reported in (E) through the calculation of the average dissimilarity index for all accessions for each pair of marker systems (see text for details). This analysis indicated that the assignments obtained with the SSRs were the most divergent from the other marker systems (DArTs and RFLPs).

This index was then used to calculate an average dissimilarity index:
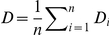
where n is the number of accessions. These calculations were performed considering two datasets built from the quantitative matrixes of assignments obtained from STRUCTURE (cf “no threshold” in Figure 1D, Table S1) and from qualitative information derived from the 0.6 membership threshold (cf “thresh = 0.6” in Figure 1D). In this case, an accession i assigned to group K with a membership superior to 0.6 was assigned a q_ik_ value of 1 for that group and 0 for the others. Accessions for which memberships were lower than 0.6 were not assigned to any genetic group.

As different marker types have different mutation rates, they are likely to reveal different patterns of genetic structure which are all of interest for understanding crop evolution. For the needs of LD extent estimation and scan for signatures of selection, we tried to determine a structure that departed as little as possible from the various patterns revealed by the different marker systems. In that purpose, we took into account the rate of assignment agreement between marker systems, the likelihood of the data for each marker system and the stability of assignments within each marker system for a given level of structure. We also used a representation obtained with a distance-based method in order to compare the obtained structure with a previously established classification scheme.

Diversity indices were calculated for each marker system within the whole CS and within the genetic groups identified. Allelic richness and private allelic richness were calculated with the rarefaction method available in HP-RARE 1.0 in order to obtain estimations based on equivalent sample size [27]. Expected heterozygosities and their standard deviations (estimated through 1000 bootstraps) were calculated with PowerMarker [28].

To compare group differentiation as estimated with the different marker systems, F statistics were calculated considering accessions assigned to the different groups with a threshold >0.6. Estimators of F*st* [29] were computed with GENETIX software [30] at the global and pairwise group levels and tested for their significance with 1000 permutations. The ability of the different marker systems to describe the structure of the accessions analysed was evaluated with the Data Resolution statistic [31]. Increasing numbers of markers were randomly sampled without replacement, by steps of 50 markers for DArTs and by steps of two markers for RFLPs and SSRs. For each number of markers, sampling was repeated 1000 times. For each sampling operation, two independent sets of half the number of markers were used to calculate two dissimilarity matrices with suitable indices according to the marker system considered (DArT: Sokal and Michener dissimilarity index, SSR: Simple matching index, RFLP: Dice index). Correlations were calculated for the two dissimilarity matrices obtained from the whole CS, considering intra or inter-group pairwise dissimilarities at the 0.6 membership thresholds.

### Linkage disequilibrium analysis

LD was estimated between genome anchored DArT markers with less than 20% missing data over the 177 accessions considered. As rare alleles induce large variances, only markers with a minor allele frequency of at least 0.05 were included in the analysis. The analyses were performed using r^2^ and D′ [32]. Since similar results were observed, only those obtained with r^2^ were presented. In addition, the advantages of r^2^ compared to other statistical parameters used to estimate the extent of LD (and more specifically D′) lie in its low sensitivity to a small sample size (low allele frequency) and easy interpretation in the context of association mapping. Indeed the r^2^ value is directly linked to the proportion of variance of the QTL (whose position is usually unknown) that will be captured by the genotyped marker [1].

Each pair of loci was categorized as unlinked (marker loci located on different chromosomes) or linked (marker loci located on the same chromosome) in six different distance intervals. Mean r^2^ values were calculated for all categories of locus pairs. In addition, the percentage of pairwise LD values beyond a threshold above which LD could not be attributed to structure only (P95 = 95 percentile of pairwise LD on different chromosomes) was defined [33]. This P95 threshold was considered as an indication of global background structure for each sample. We evaluated to what extent the LD statistics obtained on the whole CS were affected when subsets of accessions were considered. Firstly, random samples of 25, 50, 75, 100, 125 and 150 genotypes were extracted to calculate LD. For each sample size, 10 random samples were analysed in order to estimate standard errors of the estimations. Secondly, samples of the same sizes were chosen using a procedure that aims at minimizing genetic redundancy between accessions and limiting the loss of diversity. Starting from a Neighbour-Joining tree based on Sokal and Michener dissimilarity indices calculated with the DArT markers, the redundant genotypes were sequentially eliminated: i.e. at each step the pair of closest genotypes was selected and the one with the smallest external edge was removed. The procedure was iterated until the requested sample size. This procedure, called Maximum Length Subtree and referred to as MLST in this paper, is implemented in DARwin software [34], [35]. The effect of minimum allele frequency (MAF) on the extent of LD was analysed with three different minimum allelic frequency thresholds (0.05, 0.1 and 0.2). The mean *r^2^* and percentage of significant LD values increased significantly with MAF, especially for short distances (data not shown); this result was consistent with the findings of Yan *et al.* [36] in maize. Finally, LD decay within the five genetic groups harbouring sufficient numbers of accessions was also evaluated using DArT markers with a MAF larger than 0.05.

According to the LD patterns obtained in the whole CS and in the genetic groups, the numbers of markers required for genome-wide association mapping were estimated for two r^2^ thresholds (r^2^ = 0.1 and 0.3) with a non-linear regression modelling [37]. Threshold 0.1 is the minimum r^2^ value to detect associations for rather large quantitative trait loci (QTLs explaining 10% of the phenotypic variation) with a reasonable population size (300) [38] whereas threshold 0.3 was considered as the minimum value to enable detection of a QTL explaining around 5 to 10% of the phenotypic variation. The physical distances corresponding to these thresholds were obtained from the non-linear regression of LD decay with distance. The numbers of bi-allelic markers required for association studies considering these thresholds were calculated by dividing the size of the sorghum genome (736 Mb) by the LD decay distance for the entire CS and each genetic group.

### Detection of DArT F*st* outliers

Markers that present higher than expected F*st* values under neutral assumptions are candidates for divergent selection where different populations have fixed different alleles, and markers that present lower than expected F*st* values under neutral assumptions are candidates for balancing selection where diversity (heterozygosity) tend to be conserved in populations. The distribution of F*st* under neutral assumptions was calculated by two methods. Firstly, we used the infinite island model [39] implemented in LOSITAN [40]. We generated 50,000 loci for which heterozygosity and F*st* were estimated through coalescent simulations considering K populations of 50 individuals (K being the number of populations identified using STRUCTURE software and previous knowledge regarding sorghum evolution). This simulated distribution was compared to the observed F*st* values and expected heterozygosity. Markers that presented F*st* higher than the 99 percentile of neutral distribution were considered candidates for divergent selection and markers that presented F*st* lower than the 99% confidence interval were considered candidates for balancing selection. Among others, the main drawback of this approach is that all markers including those that will be candidates for adaptation are used to construct confidence interval for neutral marker F*st* distribution. Bayesian methodologies [41] were proposed to adress this limitation. The correlation of allele frequencies among demes was simulated by a multinomial-Dirichlet likelihood [42]. Two alternate models, one including selection and one excluding selection, use a reversible-jump MCMC approach to estimate the posterior probability of a given locus being under selection. We implemented 20 pilot runs of 5000 iterations, an additional burn-in of 50,000 iterations followed by 100,000 iterations with a sample size of 5000 and thinning interval of 20. Only DArT markers with log10 (Bayes Factor) equal or greater than 1 were considered, as such a threshold corresponds to a posterior probability indicative of strong evidence for selection according to Jeffreys' scale [43]. Genes located in the vicinity of the DArT markers were identified and their similarities with already characterized proteins were evaluated through a BlastP against the Swissprot database.

## Results

### Physical mapping and diversity of DArT markers

Sequencing of polymorphic DArT clones, mapping on the BTx623 genome sequence [5] and redundancy analysis led to the identification of 1410 unique loci with an average of 141 markers per chromosome (from 86 on chromosome 7 to 208 on chromosome 1) corresponding to a mean interval between markers of 670 kb on chromosome arms and 2.3 Mb in centromeric regions. The average distance between a DArT marker and the closest gene was 7.62 kb and 319 DArT markers were located within genes. Mapping of the DArT markers on the CIRAD map (mentioned in [19], Figure S1) showed almost complete colinearity between the physical and genetic maps and enabled an estimation of the recombination rates. According to this genetic map, 1 cM (Haldane mapping function) corresponds to 0.24 Mbp in euchromatin regions and 3 Mbp around centromeres; these recombination rates were in accordance with the results of Hamblin *et al.* [12] (0.254 Mbp/cM, 2–8 Mbp/cM) and Mace *et al.* [44] (0.22 Mbp/cM, 8.46 Mbp/cM).

For structure analysis, 713 informative markers (386 *Pst*I-*Ban*II and 327 MITE) having less than 10% missing data were considered, whereas 1122 markers having less than 20% missing data were used for LD analysis (Table S2). These markers were evenly distributed on the genome (Figure S1).

### Comparison of marker systems to describe genetic structure

For a comparison between markers (DArT, RFLP and SSR), 171 accessions presenting less than 10% missing data in each of the datasets were considered (Table S1). Basic statistics regarding the different markers analysed in this sample are presented in Table 1. The allelic frequency distributions differed markedly between the marker systems (data not shown). The percentage of markers harbouring a MAF below 0.1 was 6% for DArTs, 29% for RFLPs and 77% for SSRs.

**Table 1:** Genetic diversity parameters within the Core Sample (171 accessions) and within the 6 genetic groups identified.

Group^a^	N^b^	Cluster (Deu *et al.* 2006)	Race^c^	Region^d^	Total number of alleles			Allelic richness^e^			He^f^			Private allelic richness^e^		
					DArT	RFLP	SSR	DArT	RFLP	SSR	DArT	RFLP	SSR	DArT	RFLP	SSR
A	38	III & IV	D, C, CB, B	Af, ME, As	1342	142	255	1.67	2.1	3.8	0.33 (0.01)	0.30 (0.03)	0.62 (0.03)	0.01	0.11	0.76
B	28	V & VI	C, D, DC	Af	1278	121	191	1.62	1.89	3.22	0.30 (0.01)	0.28 (0.02)	0.53 (0.04)	0.01	0.05	0.48
C	22	I	G, DC	WAf	1202	99	141	1.52	1.58	2.64	0.27 (0.01)	0.17 (0.02)	0.45 (0.04)	0.01	0.02	0.34
D	10	II	Gm	WAf	928	71	118	1.26	1.18	2.66	0.16 (0.01)	0.06 (0.02)	0.42 (0.05)	0.02	0.02	0.71
E	15	VIII & IX	G	SAf, As	1175	106	142	1.52	1.75	2.86	0.25 (0.01)	0.22 (0.03)	0.49 (0.04)	0	0.06	0.33
F	24	VII	K, KC, GC	SAf	1109	92	135	1.39	1.49	2.46	0.20 (0.01)	0.16 (0.03)	0.37 (0.04)	0	0.02	0.24
UN	34	_	_	_	1404	149	290	1.80	2.29	4.07	0.39 (0.01)	0.38 (0.02)	0.66 (0.03)	0	0.05	0.52
CS	171	_	_	_	1426	172	441	_	_	_	0.41 (0.00)	0.41 (0.02)	0.68 (0.03)	_	_	_

a group number as defined in the text. UN corresponds to unassigned accessions. CS: Whole Core Sample.

b number of accessions assigned to the different genetic groups. Assignment was based on full congruence between the three marker systems (cf Table S1, column “Group Consensus Schemes”).

c races present in the group defined according to grain and spikelet morphology (Harlan and De Wet, 1972). B: bicolor, C: caudatum, D: durra, G: guinea, K: kafir, Gm: guinea margaritiferum sub-race defined according to Snowden's classification.

d Af: Africa, As: Asia, ME: Middle East, SAf: Southern Africa, WAf: Western Africa.

e allelic richness and private allelic richness computed with HP-Rare are normalized values.

f expected heterozygosity estimated with PowerMarker. Standard deviations estimated with 1000 bootstraps are shown in brackets.

**Figure 2:**
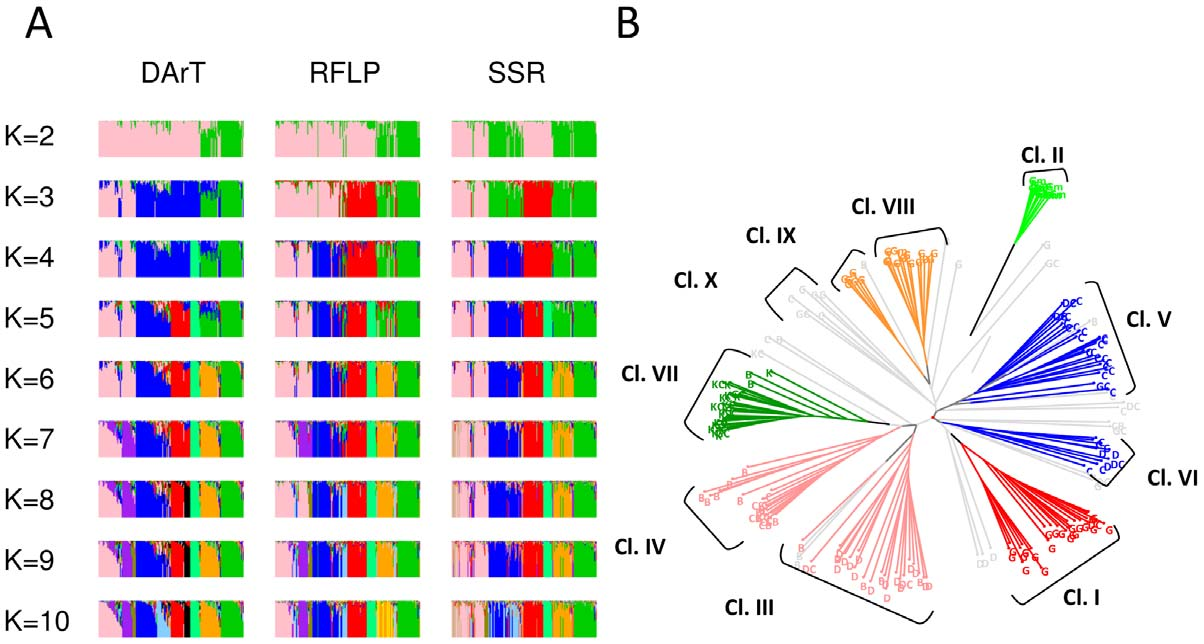
Sequential identification of the genetic groups through model-based analysis as revealed by the different marker systems and comparison of the most relevant model-based structure (K = 6) with distance-based method analysis. (A) Genome composition of accessions for different levels of structure. Each sample is represented in K dimensions, with K being the number of hypothetical genetic groups that compose the collection (K ranging from 2 to 10). Three different datasets were tested: 713 DArTs, 60 RFLPs, and 40 SSRs. Each accession on the X-axis is represented by K colours (each corresponding to a genetic group) ordered according to a decreasing genome fraction on the Y-axis. For each dataset, 171 accessions were ordered according to DArT assignments, with a decreasing proportion of genome assigned to the main groups according to STRUCTURE at K = 10. (B) Neighbour-Joining tree of the CS with colour projection of the six groups obtained with the model-based method at the 0.6 membership threshold at K = 6. The Neighbour-Joining tree is based on the genetic similarities between accessions calculated as the proportion of shared alleles of the DArT markers (Sokal and Michener modality index). The colours correspond to the genetic groups obtained at K = 6 in (A). Accessions assigned at a proportion <0.6 are coloured in grey. Group A in pink includes D and B from India, C and CB from China. Group B in blue includes C and D from Africa. Group C in red includes G from Western Africa. Group D in spring green includes Gm from Western Africa. Group E in orange includes G from Southern Africa and Asia. Group F in green includes K and KC from Southern Africa. Clusters were identified by Deu *et al.* (2006).

The number of genetic clusters present in the CS was analysed for each marker system by STRUCTURE Bayesian method. On average, tenfold more iterations were necessary to obtain a stable likelihood for SSR and RFLP datasets (10^6^) compared to DArT (10^5^). For all the marker systems, the logarithm of likelihood reached a plateau around K = 6 (Figure 1A). High ΔK values were observed at K = 2 (DArT, RFLP) and K = 4 (DArT, SSR), and lower peaks were observed at K = 7 (DArT, SSR) and K = 6 (RFLP) (Figure 1B). The rate of unassigned accessions at the 0.6 membership threshold varied with the marker systems and the number of groups (Figure 1C). For a number of groups, K, comprised between 2 and 8, SSR yielded the lowest rate of non-assignment (<10%) whereas DArT and RFLP ranged between 8 and 20%. Assignments were very stable between runs for DArT and RFLP data. For these marker systems and most K values, the average dissimilarity index between runs was low when considering quantitative assignment to groups and null when only assigned accessions at the 0.6 membership threshold were considered. The discrepancies between runs were higher for SSR, with an average dissimilarity index reaching 17% across all K values for the assigned accessions (thresh = 0.6 in Figure 1D). There were globally fewer discrepancies between DArT and RFLP than between either one and SSR (Figure 1E). An analysis of F*st* evolution for the different marker systems indicated that global F*st* stopped increasing after K = 7 for DArT, K = 8 for RFLP and continued to increase after K = 10 for SSR (data not shown). The genome plots built from the highest likelihood run of STRUCTURE outputs for each K value (Figure 2A) illustrated the groups obtained from K = 2 to K = 10 for the different marker systems. Large discrepancies between marker systems were observed for low numbers of putative genetic groups (K<4). The most striking difference was observed at K = 2, for which SSR markers failed to detect the separation of northern and southern equatorial African accessions that was detected with DArTs and RFLPs. Such difference could potentially be due to the highest mutation rate of SSR markers compared to DArT and RFLP, which could have erased the signature of ancient divergence. For higher numbers of putative genetic groups, although the congruence between the marker systems was better, slight differences remained. At K = 10, a good congruence of the DArT and RFLP systems were observed, the only differences concerning the split of the guinea group from West Africa in two sub-groups (red and black) with the DArTs and the alternative split of the guinea group from South Africa in two sub-groups (orange and yellow) with the RFLPs. At K = 15, these 4 groups were characterized for each marker system (data not shown), as well as a group from the African Great Lake already mentioned by Deu *et al.* (2006) [6]. The differences were much stronger with the SSR markers. The best congruence of assignments between marker systems was obtained at K = 6. Within this consensus scheme, 80% of the accessions were assigned to a genetic group (137 among 171 accessions). Unassigned accessions included 75% (24) of accessions phenotypically identified as pure whereas 25% (8) corresponded to intermediate types. However, the low proportion of intermediate types in the collection analyzed (28 accessions) did not allow us to draw clear conclusions regarding the predictive power of the different marker systems for assignment and de-novo detection of admixed accessions. Within the consensus scheme, group A (pink) was composed predominantly of durra and bicolor types from India (22%) and eastern Africa (17%), and caudatum and caudatum-bicolor types from China (23%). Group B (blue) comprised caudatum and durra types, as well as intermediate types from Africa. Group C (red) mainly comprised guinea types from western Africa. Group D (spring green) exclusively comprised guinea margaritiferum types from western Africa. Group E (orange) comprised mainly guinea types from southern Africa and Asia. Group F (green) predominantly comprised kafir and kafir-caudatum types from southern Africa (80%). Projection of these groups on the Neighbour-Joining tree based on the DArT markers revealed a good correspondence between the Bayesian approach implemented in STRUCTURE and distance based methods (Figure 2B). It is also interesting to note that the consensus scheme observed at K = 6 and the Neighbour-Joining tree obtained with DArT markers were highly congruent with the structure identified by Neighbour-Joining in Deu *et al.* [6] with RFLP markers (Table 1). The global differentiation between the 6 groups, as measured by F*st*, appeared comparable between DArTs (0.42, p<0.001) and RFLPs (0.48, p<0.001) and lower with SSRs (0.29, p<0.001). Pairwise F*st* between groups were accordingly lower with SSR (Table 2), even though they were closely correlated with those obtained with DArTs (r = 0.95) and RFLPs (r = 0.93), thereby confirming the higher diversity level within each group as described with SSR (Table 1). Groups A and B exhibited greater gene diversity, allelic richness and private SRR allelic richness compared to groups E and F.

**Table 2:** Pairwise differentiation (Fst) between the six genetic groups as obtained with three different marker systems (DArT, RFLP, SSR).

Group1	Group2	DArT_F*st*	RFLP_F*st*	SSR_F*st*
Whole Core Sample		0.42	0.48	0.29
A	B	0.26	0.28	0.18
A	C	0.35	0.41	0.25
A	D	0.51	0.54	0.36
A	E	0.36	0.36	0.22
A	F	0.43	0.47	0.26
B	C	0.31	0.42	0.29
B	D	0.55	0.59	0.39
B	E	0.32	0.46	0.21
B	F	0.45	0.53	0.29
C	D	0.59	0.67	0.45
C	E	0.41	0.49	0.33
C	F	0.53	0.64	0.38
D	E	0.62	0.67	0.43
D	F	0.70	0.71	0.52
E	F	0.36	0.40	0.28

Genetic differentiations were calculated among the genetic groups considering only those accessions assigned with a membership > = 0.6. For DArT, RFLP and SSR markers, F*st* values (Weir and Cockerham, 1984) were computed with GENETIX.

### Data resolution

**Figure 3:**
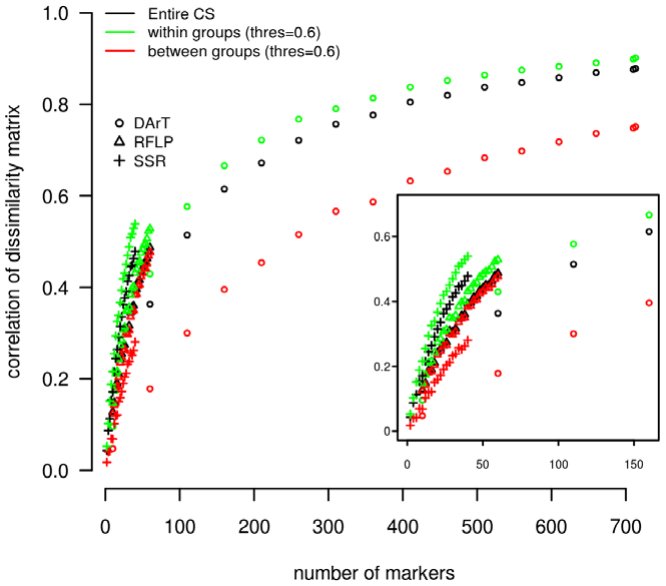
Ability of the marker systems to describe the genetic structure of the sorghum Core Sample. Total, intra- and intergroup accession dissimilarity correlations obtained with an increasing number of the three different marker systems (DArT, RFLP, SSR) were computed according to the data resolution statistic developed by Van Hintum [31]. An enlargement of the graph for marker numbers comprised between 0 and 150 is provided at the bottom right-hand side of the figure. For the whole CS, the description of diversity can only be considered saturated for DArT markers. An analysis of the dissimilarity correlations at the intra- and intergroup levels indicated that SSR markers were the least efficient in describing the intergroup structure.

Data resolution analysis for the three marker systems indicated that the information could be considered saturated only for DArT markers for which correlations between dissimilarity matrices reached a plateau (r = 0.8) at around 300 markers. For RFLP and SSR, correlations between dissimilarity matrices only reached 0.49 and 0.48 respectively, suggesting that an increase in marker number would be necessary to obtain a stable representation of genetic diversity (Figure 3). According to the identification of a consensus scheme involving 6 genetic groups, data resolution of the different marker types were evaluated at the intra- and intergroup levels at the 0.6 membership threshold. Whatever the marker systems, the correlations were higher at the intragroup than at the intergroup level (DArTs: 0.90 vs 0.75, RFLPs: 0.53 vs 0.48 and SSRs: 0.54 vs 0.28). Although these differences were limited for DArTs and RFLPs, the data resolution for SSRs at the intra- and intergroup levels were highly different, suggesting that intergroup dissimilarities remained very unstable with the set of SSRs used in this study.

### Linkage disequilibrium

#### Global LD decay

**Figure 4:**
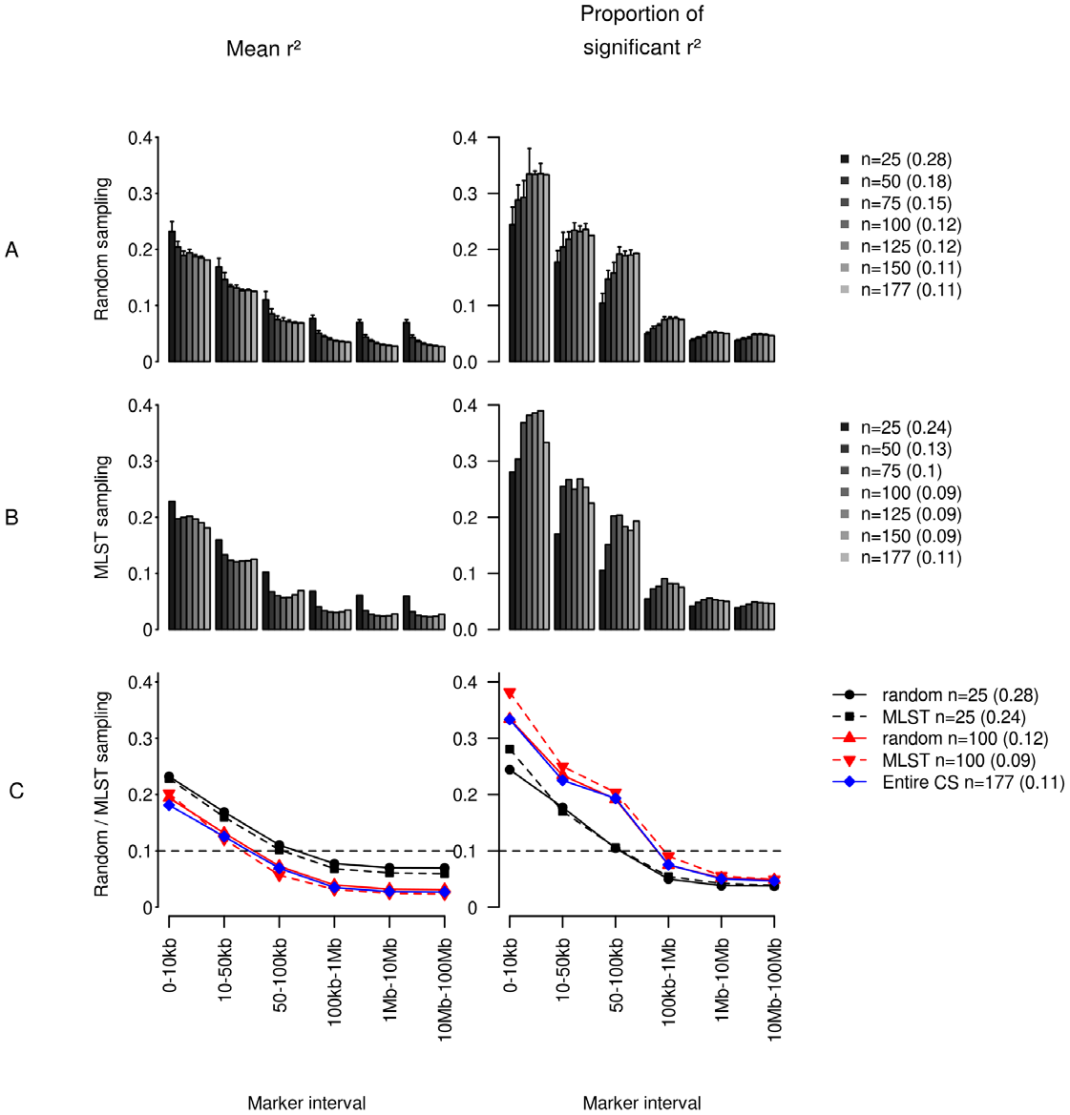
Evolution of linkage disequilibrium for different sample sizes and distances. Mean r^2^ and the proportion of significant pairwise r^2^ (i.e greater than P95) were computed for the CS and for subsets of accessions ranging from 25 to 150. Accessions were sampled using two strategies. Firstly, random samples of 25, 50, 75, 100, 125 and 150 genotypes were extracted to calculate LD statistics. For each sample size, 10 random samples were analysed in order to estimate standard errors of the estimations (A). Secondly, a procedure designed to define subsets of genotypes minimizing their redundancy and limiting the loss of diversity was used (MLST reported in (B)). A comparison of these two sampling approaches for three sample sizes is provided in (C) and indicates that, with both strategies, mean r^2^ was overestimated for all distance classes in small samples (n = 25). The proportion of significant pairwise r^2^ was always higher with the MLST, compared to the random approach, especially for small distances and small sample sizes, highlighting the efficiency of this algorithm in providing a more accurate local LD estimation through the reduction of background LD. After correcting for background structure, mean r^2^ decreased with distance from 0.2 (for the 0–10 kb distance class) to 0.02 and stabilized after 100 kb (B). Contrary to random sampling, an increase in mean r^2^ (B) was observed with the MLST approach when sample sizes greater than 100 accessions were considered, suggesting that redundancy, and thus background structure, was introduced after this sample size. These results indicate that, for the CS, a sample size of 100 accessions carefully selected to avoid redundancy and maximize diversity would be an optimized sample size for LD estimation.

A total of 1122 physically anchored DArT markers genotyped on 177 accessions were considered for LD analysis. LD statistics for six classes of interval distances are summarized in Table 3 and Figure 4. For the CS, mean r^2^ decreased from 0.18 (for the 0–10 kb interval) to 0.03 (for the 100 kb–1 Mb interval), stabilizing at 0.03 after 1 Mb. The proportion of significant r^2^ values (i.e. independent of background LD) decreased from 33% to 8% in the same intervals, stabilizing at 5% after 1 Mb.

**Table 3:** Evolution of linkage disequilibrium with physical distance in the Core Sample.

Distance	Npairs^a^	Mean (r^2^)	SD (r^2^)	Proportion of significant r^2^ b
0–10 kb	60	0.18	0.29	0.33
10–50 kb	151	0.13	0.25	0.23
50–100 kb	176	0.07	0.11	0.19
100 kb–1 Mb	2989	0.03	0.07	0.08
1 Mb–10 Mb	21426	0.03	0.05	0.05
10 Mb–100 Mb	41839	0.03	0.05	0.05

a number of pairs of markers.

b proportion of r^2^ values greater than the P95 threshold (this threshold corresponded to r^2^>0.11 for the whole Core Sample).

#### LD and sample size: comparative analysis of two sampling strategies

Random sampling of different sample sizes (n = 25, 50, 75, 100, 125, and 150) with 10 repetitions was carried out to calculate LD statistics (Figure 4A). As observed in Figure 4A, mean r^2^ was higher for small samples (n = 25 to 75) for all distance classes and then stabilized for larger sample sizes (n≥100). The P95 threshold decreased from n = 25 (0.28) to n = 100 (0.12) and then stabilized (0.11) leading to an increase in the proportion of significant r^2^ values (from 24% for n = 25 to 33% for n = 100). This suggests that when n<100, LD for unlinked markers was overestimated (strong background LD effect) and LD between linked markers was underestimated.

MLST-based samples of size 25, 50, 75, 100, 125 and 150 yielded lower P95 thresholds for significant LD (from 0.24 to 0.09 when sample size increased) compared to those obtained by random sampling (from 0.28 to 0.11) (Figure 4B). A comparison of LD correlations between the CS and sub-samples obtained with the two sampling strategies (Figure S2) indicated that correlations increased much faster with samples of increasing sizes obtained through the random strategy than with the MLST algorithm. These observations come from the fact that the random sampling strategy maintained the redundancy available in the whole CS, with the groups most represented in the CS contributing more than those that were under-represented, leading to biased LD estimates. The comparison of the two strategies provided in Figure 4C indicates that mean r^2^ was overestimated for all distance classes in small samples (n = 25) for both strategies. However, the proportion of significant r^2^ values was always higher with the MLST strategy than with the random strategy (Figure 4C), especially for short distances and small sample sizes; this observation denoted the efficiency of this algorithm in estimating local LD by reducing background LD. Despite the correction provided by the MLST algorithm, the percentage of significant LD values remained underestimated for n = 25–50 compared to higher sample sizes (Figure 4B). Unlike random sampling, for which almost continuous increases in mean r^2^ and in the proportion of significant r^2^ were observed until n = 150–177, the MLST strategy featured a maximum LD resolution for n = 100 with a slight decrease above that, with higher sample sizes probably leading to a redundancy between the accessions. Consequently, a set of 100 accessions sampled with the MLST strategy provided the most accurate LD estimates for the CS. Considering this “Optimized 100 MLST set” (100 accessions sampled with MLST), it appeared that mean r^2^ decreased with distance from 0.2 to 0.02 and stabilized after 100 kb. The proportion of significant values decreased concomitantly from 38 to 21% between the same intervals, but continued to slowly decrease for larger inter-marker distances (Figure 4B). Estimations of the number of markers required for association mapping in the CS are provided in Table 4.

**Table 4:** Number of markers required for association mapping studies.

Group	a = N_e_ρ^a^	Achieved convergence tolerance^b^	Number of markers (r^2^ = 0.1)	Number of markers (r^2^ = 0.3)
CS	3.63E-04	8.01E-06	>100 000	>350 000
A	4.03E-05	6.76E-06	>13 000	>50 000
B	5.22E-05	7.01E-06	>17 000	>65 000
C	1.17E-05	9.73E-06	>3500	>15 000
E	6.22E-06	7.95E-06	>2000	>8 000
F	1.41E-05	7.56E-06	>4500	>18 000

a Decay of LD with physical distance were fitted according to Ohta and Kimura (1969) [37] model: E (r^2^_ij_) = 1/(1+4Neρd_ij_ρ). Where, r^2^_ij_ stands for the LD value between markers i and j, Ne for the effective population size, ρ for the per base recombination rate and d_ij_ for the distance in base pairs between markers i and j. The analysis was performed with the nls function in R. The composite parameter a = Neρ was estimated for the whole CS and the different genetic groups.

b Achieved convergence tolerance obtained with the nls function in R.

#### LD in subgroups of different origins

In addition to the analysis of LD evolution in the whole CS, LD decay variability was estimated in the different genetic groups (excluding group D because of its small size with 11 accessions). Mean r^2^ values (Figure S3) were quite different across genetic groups. The thresholds of significant LD values were comparable (0.3), but higher than in the whole CS (0.11) and the “optimized 100 MLST” set (0.09). Percentages of significant values were also variable between groups, with the most diverse groups, A and B, showing lower LD (r^2^ = 0.1 at 100 kb and fewer than 10% of marker pairs harbouring significant LD) and group E, which was the least diverse and the most recent, showing higher LD (r^2^>0.2 at 100 kb and more than 25% of marker pairs harbouring significant LD). These results show that variable extents of LD are expected within the different genetic groups and highlight the fact that different marker densities will be required if association studies are planned in the whole CS or in the different genetic groups (Table 4).

### DArT outlier detection

A total of 1122 DArT markers were tested for evidence of selection through an outlier detection approach. The consensus scheme involving the six previously identified diversity groups was used for the differentiation-pattern analysis. The 99% confidence interval obtained with the finite island model implemented in LOSITAN led to the detection of 190 outlier loci, of which 11 loci were candidates for diversifying selection and 179 loci were candidates for balancing selection (Table 5). BAYESCAN analysis led to the detection of 23 (2.5%) outlier loci with a log 10 (Bayes factor) greater than 1.0, of which 9 were consistent with the evidence of diversifying selection and 14 corresponded to balanced polymorphism. Among these 23 loci, three remained outliers when considering log 10 (Bayes factor) greater than 1.5 and only one when considering log 10 (Bayes factor) greater than 2, with these three markers corresponding to loci under diversifying selection. The 14 outliers harbouring evidence of balancing selection with BAYESCAN were also detected with LOSITAN, whereas only one locus was common for the diversifying selection pattern.

**Table 5:** DArT markers presenting evidence of selection.

S^a^	M^b^	DArT Gene bank accession^c^	BAYESCAN F*st* d	LOSITAN F*st* e	Chr/Closest gene	Dis^f^	Protein Accession	Protein description^g^	E-Value	Similarity %	Biological Process^h^
b	b+l	FI849516	0.11 (1.01)	0.02 (3.E-04)	2/Sb02g005150	0.0	Q851R2	Argonaute MEL1	0.E+00	82	R
b	b+l	FI847862	0.10 (1.13)	0.00 (3.E-05)	2/Sb02g035650	0.7	Q6ZL42	Probable histone H2A.2	7.E-37	100	DR
b	b+l	FI849537	0.11 (1.08)	0.02 (2.E-04)	2/Sb02g037020	0.0	Q8RWS8	Pentatricopeptide repeat-containing protein	0.E+00	79	R
b	b+l	FI849079	0.11 (1.01)	0.01 (0)	3/Sb03g001840	0.0	Q94F40	GDSL esterase/lipase	4.E-66	58	o
b	b+l	FI847745	0.10 (1.18)	−0.02 (0)	4/Sb04g024000	2.0	Q8GWP0	Transcription factor MYB39	4.E-56	86	DR
b	b+l	FI849457	0.09 (1.38)	−0.02 (0)	5/Sb05g026780	0.0	No hit	_	_	_	_
b	b+l	FI848743	0.10 (1.25)	−0.01 (0)	5/Sb05g027033	0.1	Q39214	Disease resistance protein RPM1	5.E-36	43	D
b	b+l	FI848764	0.10 (1.11)	−0.01 (0)	7/Sb07g003170	69.9	Q2V9B0	Transcription factor MYB1R1	2.E-25	57	S
b	b+l	FI849152	0.11 (1.00)	0.00 (0)	7/Sb07g026000	1.1	Q74ZH9	Glycerophosphodiester phosphodiesterase	1.E-14	40	o
b	b+l	FI849546	0.09 (1.31)	−0.02 (0)	9/Sb09g015250	0.0	Q9LRR4	Putative disease resistance RPP13-like protein 1	5.E-59	46	D
b	b+l	FI849032	0.10 (1.30)	−0.01 (2.E-05)	9/Sb09g021330	0.0	Q0WP01	Probable peptide/nitrate transporter	2.E-151	72	o
b	b+l	FI849030	0.10 (1.25)	−0.01 (0)	9/Sb09g028567	0.7	No hit	_	_	_	_
b	b+l	FI849109	0.10 (1.10)	−0.02 (0)	10/Sb10g023860	0.0	Q9LY87	E3 ubiquitin-protein ligase RGLG2	0.E+00	88	M
b	b+l	FI847767	0.10 (1.08)	−0.01 (0)	10/Sb10g025040	2.0	No hit	_	_	_	o
d	b	FI847680	0.55 (1.14)	0.72 (0.96)	1/Sb01g019560	7.4	Q86TV6	Tetratricopeptide repeat protein 7B	6.E-13	62	o
d	b	FI848942	0.58 (1.80)	0.72 (0.97)	2/Sb02g026890	3.9	No hit	_	_	_	_
d	b	FI848913	0.54 (1.04)	0.75 (0.98)	2/Sb02g037690	1.0	Q920Q6	RNA-binding protein Musashi homolog 2	9.E-47	54	DR
d	b	FI847959	0.53(1.13)	0.68 (0.94)	3/Sb03g045960	2.6	O81117	Cytochrome P450 94A1	5.E-88	56	D
d	b	FI847986	0.61 (2.47)	0.74 (0.98)	5/Sb05g007936	99.3	Q6H8D5	Beta'-coat protein 2	2.E-21	52	o
d	b	FI848529	0.54 (1.04)	0.52 (0.75)	6/Sb06g032030	1.9	P13240	Disease resistance response protein 206	3.E-04	50	D
d	b	FI847989	0.58 (1.13)	0.58 (0.79)	8/Sb08g019196	6.0	Q7XWS7	Formin-like protein 12	7.E-159	90	o
d	b	FI849519	0.57 (1.45)	0.61 (0.89)	8/Sb08g020740	0.3	Q2QMH2	Protein ROOT HAIR DEFECTIVE 3 homolog 1	0.E+00	84	M
d	b+l	FI849491	0.61 (1.69)	0.78 (1.00)	4/Sb04g000390	0.7	P16157	Ankyrin-1	3.E-13	54	o
d	l	FI849130	0.35 (−0.81)	0.74 (0.99)	1/Sb01g046750	0.0	Q0DV28	Armadillo repeat-containing kinesin-like protein 1	0.E+00	83	o
d	l	FI847843	0.44 (−0.10)	0.93 (1.00)	2/Sb02g033230	3.5	P40691	Auxin-induced protein PCNT115	2.E-132	86	o
d	l	FI848068	0.39 (−0.28)	0.80 (1.00)	3/Sb03g006130	6.2	P12863	Triosephosphate isomerase	2.E-128	97	o
d	l	FI849137	0.39 (−0.47)	0.91 (1.00)	3/Sb03g026270	0.3	Q8LBB2	SNF1-related protein kinase regulatory subunit gamma 1	6.E-42	62	o
d	l	FI848736	0.56 (0.94)	0.88 (1.00)	4/Sb04g036180	0.6	Q8VEH6	Cobalamin synthase W domain-containing protein 1	3.E-56	56	o
d	l	FI849358	0.38 (−0.55)	0.89 (1.00)	5/Sb05g000450	4.8	Q00610	Clathrin heavy chain 1	0.E+00	77	o
d	l	FI847787	0.51 (0.38)	0.90 (0.99)	6/Sb06g003220	11.7	Q9SWG3	Protein FAR-RED IMPAIRED RESPONSE 1	4.E-14	40	F
d	l	FI849042	0.48 (0.36)	0.80 (0;99)	6/Sb06g017640	0.0	No hit	_	_	_	_
d	l	FI848273	0.39 (−0.46)	0.91 (1.00)	10/Sb10g009520	9.5	Q84L30	Putative DNA repair protein RAD23-4	3.E-73	77	DR
d	l	FI849518	0.58 (0.56)	0.98 (0.99)	10/Sb10g020500	3.3	Q9FLG1	Beta-D-xylosidase 4	0.E+00	59	CW

a Type of selection detected, b stands for balancing selection whereas d stands for diversifying selection.

b Method used to identify the considered locus, b stands for BAYESCAN (log10(Bayesfactor)>1, l for LOSITAN (CI>0.99) and bl corresponds to loci detected with both methods.

c The DArT marker name corresponding to the genebank accession provided can be found in Table S2 (Marker_Name column).

d The F*st* value obtained with BAYESCAN, the log 10 (Bayes factor) is provided in brackets.

e The F*st* value obtained with LOSITAN, the Pvalue corresponding to P(Simul Fst<sample Fst) is provided in brackets.

f Distance between DArT marker and the closest gene in kb.

g Protein presenting the highest similarity with the gene located in the vicinity of the DArT marker as detected by a BlastP against the Swissprot database, the Evalue and Similarity percentage at the amino acid level are provided in the following columns.

h Biological process in which the gene is involved: D: Disease resistance, S: Abiotic stress resistance, F: Flowering regulation, CW: Cell wall establishment, M: plant morphology, R: reproduction, DR: DNA repair or Regulation of transcription or Regulation of translation, o: others.

LOSITAN enabled the detection of 179 loci (16% of the markers) with evidence of balancing selection. This result is congruent with Narum and Hess [45] who observed that LOSITAN led, under several different demographic scenarios, to the detection of elevated levels of false positives in the case of balancing selection (14–30%). Consequently, while keeping markers detected by LOSITAN and/or BAYESCAN (19 outliers) for diversifying selection, we only kept markers detected using both methods (14 outliers) for balancing selection. Nine DArT outliers were located in annotated genes, whereas 17 were located at less than 5 kb, and 5 were located at distances ranging from 5 to 11.7 kb from previously identified genes. Of the 31 genes located in a 12 kb window from a DArT marker, 25 displayed homology with characterized proteins (E values greater than 1.10^−10^) (Table 5).

## Discussion

In this study, the properties of a set of physically anchored DArT markers, in relation with the description of the genetic structure of a worldwide sorghum collection, were compared to two other marker systems. This resource also enabled to refine the extent of linkage disequilibrium in the CS. Lastly, the genome coverage reached with DArT markers enabled the identification of genomic regions harbouring signatures of selection that are likely to be of adaptive interest.

### Genetic structure as revealed by different markers

Sorghum diversity has been recently analysed based on two large panels [6], [8] meant to be representative of worldwide diversity and harbouring contrasting compositions in terms of the geographical and racial origins of the accessions. These two collections were analysed with relatively limited numbers of markers and the stability of the genetic structures identified was not clearly assessed.

Our study highlighted the relevance of the newly developed DArT markers to describe the global genetic structure of worldwide sorghum in comparison with other genetic markers. RFLP and DArT markers provided more stable assignments of the accessions compared to SSR markers and were globally more congruent than with SSRs. Moreover, a data resolution analysis applied to the three marker datasets [31] clearly indicated that the information could be considered saturated only for the set of DArT markers. The larger number of DArT markers certainly contributed to that performance. Using the suggestion made by Laval *et al.* [46] that m loci with k alleles per locus on average are equivalent to m*(k-1) bi-allelic markers, our SSR data would be equivalent to 40*10 = 400 and our RFLP dataset to 60*1.9 = 114 bi-allelic markers. The data resolution capacity of 114 DArT markers appeared slightly higher than that of our RFLP dataset and that of 400 DArT markers was markedly higher than that of our SSR data set. While RFLPs and DArTs may have comparable grounds in terms of sequence variation, the properties of SSR variation, with high mutation rates and homoplasy, are likely to hide some ancient phylogenetic signals. For instance, the divergence between the northern and southern equatorial accessions was not directly detected with this marker system. While the global genetic differentiation estimates (F*st*) were always lower for SSRs, pairwise F*st* between the considered genetic groups were highly correlated between marker systems, as often observed in a range of species [47]–[50]. This pattern fitted well with the expectations developed through simulation work [51] in the case of a low migration rate among highly differentiated populations; it reinforced the view that sorghum domestication involved early differentiation of the main genetic groups due to geographical isolation and low gene flow in relation to farmer practices [52], [53].

The consensus scheme with six groups was consistent with the racial and geographical pattern highlighted by previous analyses of worldwide sorghum diversity [6]–[9]. Although it is clear that higher levels of structure are biologically meaningful (Figure 4A), and that a larger sampling would undoubtedly allow refining our understanding of sorghum genetic structure, it is interesting to note that this scheme has been found to be accurate in decreasing the proportion of false positive tests (70%) due to population structure in association studies [21]. In addition, this genetic structure actually reflects different steps in the domestication history of sorghum, groups A and B being representative of the original centre of domestication (also in accordance with high levels of diversity observed for the different marker systems), whereas the remaining groups can be considered as secondary centres of diversification (West Africa: C and D, Southern Africa: E and F).

### Linkage disequilibrium

Current knowledge on LD in *Sorghum bicolor* rests on the study of Hamblin *et al.* [12] on 24 landraces and 9 wild sorghums chosen to maximize the diversity and capture the evolutionary history of *S. bicolor* studied across six unlinked regions of 40–100 kb. The development of physically anchored DArT markers evenly spaced on the sorghum genome and genotyped on 177 accessions representative of worldwide cultivated sorghum brings about complementary observations, in terms of accession sub-sampling and marker density requirements for association mapping within the whole population and the various genetic groups.

We showed how small sample sizes lead to an overestimation of background LD and to a concomitant underestimation of physical LD, as was observed in maize [36]. However, we also showed how a sampling strategy designed to minimize the redundancy between accessions without losing diversity (Maximum Length SubTree) helped diminish background LD and retrieve less biased LD estimates. It also showed that an optimum sampling size to efficiently evaluate LD within this sorghum worldwide sample would be around 100 accessions carefully chosen with the MLST algorithm.

As mentioned previously by Hamblin *et al.* [12], LD in sorghum largely decays by 10–15 kb. Yet our results also indicated that significant LD could be found at much longer distances. We found significant LD for about 20% of the pairs of markers in the 50–100 kb range. We also found some regions with significant LD spanning between 0.5 and 2 Mbp on each chromosome, 5 Mbp on chromosome 7 and more than 12 Mbp on chromosome 10; these cases of long range LD probably reflect the occurrence of introgression from distant genetic groups or selection events.

The extent of LD within the different genetic groups appeared variable. The most diverse groups (A and B) exhibited rapid LD decay in contrast with the less diverse groups E and F. These results are consistent with the current evolutionary scenario for cultivated sorghum: genetic groups from East Africa (which contribute to the main share of groups A and B) correspond to the primary centre of domestication, with high diversity and many private alleles, whereas the genetic groups from southern Africa (E and F) correspond to more recent secondary centres of domestication that have been strongly affected by genetic drift, leading to lower diversity and few private alleles.

Although it is clear that more markers will be required to obtain accurate estimates of the marker density required for association mapping especially to take into account within chromosome LD variability, we roughly estimated that more than 100,000 markers may be required for whole genome scans within the CS if an r^2^ threshold of 0.1 is considered and 350,000 for a threshold of 0.3.

Within the different genetic groups considered, these requirements varied between 2000 (8000) for group E, which is one of the least diverse, to 17,000 (65,000) for group B, which encompasses wide diversity. It is likely that the required marker density estimated from our CS will also stand for the converted panel analysed by Casa *et al.* [8], and Brown *et al.* [9], as it also relies on extremely wide diversity. In comparison with other monocotyledonous species of agronomic importance, the LD decay pattern observed for sorghum (r^2^>0.1 for intermarker distance comprised between 10 and 50 kb) lies between maize (extent of LD comprised between 1 and 10 kb depending on chromosomes [36]) and rice (extent of LD probably over 500 kb in *Oryza sativa japonica* [54]). These results highlight the potential merits of a comparative analysis between these three species, for traits of general interest for which QTLs have been detected in the same regions, each one providing different levels of accuracy enabling progressively fine mapping of the gene of interest (assuming that the different species share the same genetic architecture).

### Signatures of selection

The search for targets of selection in sorghum has so far mainly relied on the analysis of small panels of accessions characterized with limited numbers of loci [11], [13]–[17]. The development and genotyping of a large set of physically anchored DArTs offered the opportunity to detect genomic regions affected by selective events. Given the genetic structure of sorghum and its likely coincidence with some adaptive processes, the search for loci exhibiting higher than expected differentiation between groups, as well as loci that maintain high diversity levels within the different groups, will lead to candidate genes for positive or balancing selection, respectively.

Whilst this first genome scan for selection in cultivated sorghum can be considered preliminary, given its density, it led to some encouraging results. Of the 33 outlier loci identified, 26 were located at less than 5 kb from annotated genes and 9 were located in genes; this preferential location of the outlier markers in the vicinity of annotated genes provides the first support for their putative relevance. In terms of gene function, the most represented biological processes were related to disease resistance (4 genes) and DNA repair, transcription and translation regulations (4 genes) (Table 5).

Marker sPb-9936 (FI849546) was located within gene Sb09g01525, corresponding to a putative disease resistance RPP13-like protein 1 (At3g14470) and marker sPb-1794 (FI848743) was located at 120 bp from Sb05g027033, corresponding to a putative disease resistance protein RPM1. For both genes, the evidence of balancing selection detected in this study corroborates observations reported in other species (RPM1: [55]–[57], RPP13: [58]).

**Figure 5:**
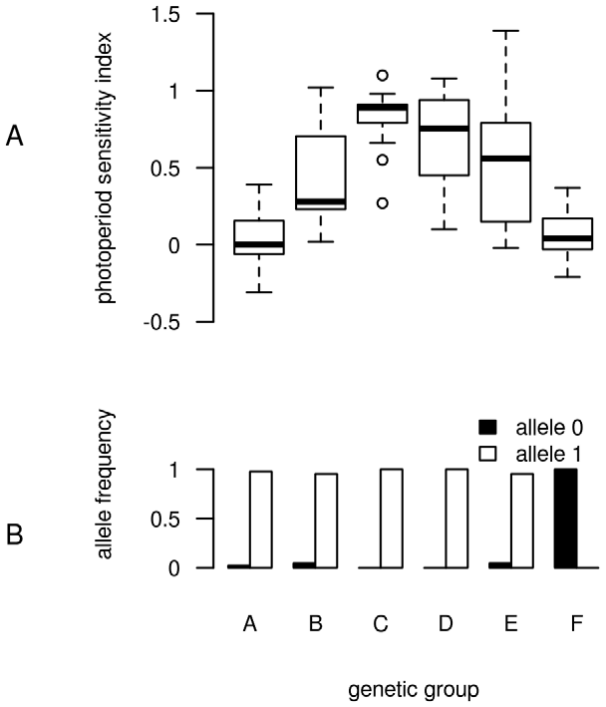
Concomitant variability of the photoperiod sensitivity index with the allelic frequency of the DArT SbMITE-188058. The data for the photoperiod sensitivity index (Kp, which corresponds to the decrease in the duration of the vegetative phase between two sowing dates) presented in (A) were obtained from Clerget *et al.* [70] who used the same collection of accessions. Kp varies from 0 for photoperiod-insensitive varieties, which do not change the duration of their vegetative phase with the sowing date, to 1.0 for the most strongly photoperiod-sensitive varieties which maintain their calendar date of flowering constant by reducing the duration of their vegetative phase. A total of 136 accessions with a membership coefficient greater than the 0.6 threshold were considered. These accessions corresponded to 40 accessions from group A, 27 from group B, 21 from group C, 11 from group D, 17 from group E and 22 from group F. An Anova analysis indicated highly significant differences between the genetic groups (p value<2.2e-16) with genetic group F harbouring a low photoperiod sensitivity index. An analysis of the variability in the allelic frequency of the DArT marker SbMITE-188058 (FI847787) located at 11.7 kb from a homologue of the FAR-RED IMPAIRED RESPONSE 1 protein isolated in *Arabidopis thaliana* (B) highlighted the specificity of group F, suggesting a potential role of this gene in the genetic control of variability in photoperiod sensitivity.

Marker SbMITE-188058 (FI847787) was located at 11.7 kb from a homologue of the FAR-RED IMPAIRED RESPONSE 1 (FAR1) protein isolated in *Arabidopis thaliana* and displayed evidence of diversifying selection. FAR1 is a transposase-derived transcription factor that mediates phytochrome A responses to far red light [59]–[62]. Such a mechanism is directly involved in the control of flowering and growth. In *Picea abies*, Clapham *et al.* [63] showed a clinal variation in requirement for far red light to initiate budset depending on the latitudes of origin of the populations. In our study, group F, made up exclusively of kafir accessions from South Africa, was the most differentiated from the others in terms of allelic frequencies at this locus (Figure 5B). In addition, these accessions originate from the southernmost part of the sorghum distribution area and display a very low photoperiod sensitivity index (Figure 5A). Based on these observations, it is tempting to propose the contribution of the genomic region encompassing this sorghum homologue of FAR1 protein as a potential driver of adaptation to southern latitudes through, at least in part, lower photoperiod sensitivity. However, an increase in the frequency of neutral alleles can also be observed at the edges of a range expansion leading to the same signal that would be observed in the case of positive selection [64]. Such a scenario cannot be ruled out in our case, as Southern African populations indeed correspond to the extreme range of sorghum distribution and because low gene flow occurs with other genetic groups. As proposed by Mariac *et al.* [65], we are planning to test the putative involvement of this genomic region in the genetic control of photoperiod sensitivity taking an association mapping approach.

Marker sPb-9731 (FI849518), displaying a signature of diversifying selection, was located at 3.3 kb from a homologue of a Beta-D-xylosidase 4 gene isolated in *Arabidopis thaliana* (AtBXL4) and involved in cell wall establishment [66]. The most differentiated group was group D which encompassed guinea margaritiferum from western Africa. These accessions present singular cell wall characteristics compared to the others, displaying high similarities with wild sorghum accessions (*Sorghum bicolor* ssp verticilliflorum) in terms of fibre, lignin, cellulose and hemicellulose content (personal communication, David Pot).

Markers Pb-9432 (FI849491) and sPb-9688 (FI849516) finely tagged two genes (Ankyrin-1 and Argonaute MEL1 respectively) which, in other plants (*Arabidopsis thaliana* and white spruce), have been reported to display deviations from neutral expectations, although these studies did not conclude on the same type of selection [67], [68]. These diverging results are not unexpected, as the type of selection acting on a gene can be different between species or environments.

All in all, our results often contribute to an array of independent studies which provided congruent information underlying the putative importance of the genomic regions identified in the genetic control of key adaptive traits.

### Conclusions

In this study, characterization of a large set of physically-anchored DArT markers on a germplasm sample representative of worldwide cultivated sorghum showed its relevance for describing genetic structure and for exploring genome-wide applications. Although a higher marker density will be required to accurately describe LD variability at the whole genome scale, this study contributed to reach a better image of LD decay in sorghum and provided the first guidelines for the marker densities required for association mapping. We showed that optimization of population composition through the MLST algorithm is an efficient way of obtaining LD estimates that are less affected by population structure. The set of markers developed also enabled the identification of genomic regions of potential adaptive interest through an evolutionary based strategy, which constitutes a complementary approach to association studies in identifying the genetic factors affecting variability in traits of interest. This study contributes to the genomic resources available for the sorghum community, as most of the accessions analysed also belong to the Reference Set that has been developed under the Generation Challenge Programme (http://test1.icrisat.org/sorghum/Sorghum_Reference.htm).

## Supporting Information

Figure S1 **Collinearity of physical and genetic maps.** This figure illustrates the genome coverage of the physical (SBI) and genetic (LG) maps of sorghum with the DArT genotyping tool. The genetic map (CIRAD Map mentioned in Mace [19] *et al.* 2009) includes 507 DArTs, 180 SSRs and 52 RFLPs. The physical map includes 1346 non-redundant DArTs (1412 loci), with 436 in common with the genetic map and 138 SSRs. Ninety-eight percent of the markers are collinear. *Pst*I-*Ban*II DArT markers are indicated in green, MITE DArT markers are indicated in red and SSR and RFLP markers are indicated in grey.
(TIF)

Figure S2 **Correlations between linkage disequilibrium estimates obtained either with the Random or the MLST sampling strategies and the whole Core Sample.** The increase in correlations with sample size was much faster with random sampling than with the MLST strategy. These observations probably come from the fact that in the random sampling strategy, the redundancy available in the whole CS was maintained in the sub-samples and the groups that are the most represented in the CS contributed more than those that are under-represented, leading to the same biases as those observed with the whole CS.
(TIFF)

Figure S3 **Evolution of linkage disequilibrium in the different genetic groups.** Mean r^2^ (A) and the proportion of significant pairwise r^2^ (i.e. greater than P95) (B) were computed for the different genetic groups. Mean r^2^ were quite different between the genetic groups. Percentages of significant values were also variable between groups, with groups A and B, which were the most diverse, showing lower LD (r^2^ = 0.1 at 100 kb and less than 10% of marker pairs harbouring significant LD) and group E, which was the least diverse and the most recent, showing higher LD (r^2^>0.2 at 100 kb and more than 25% of marker pairs harbouring significant LD).
(TIFF)

Table S1 **List of accessions used in this study.**
(XLSX)

Table S2 **DArT marker information.**
(XLSX)
