# Supplementary figures and images for: Genetic Structure, Linkage Disequilibrium and Signature of Selection in Sorghum: Lessons from Physically Anchored DArT Markers
Source: PLoS One. 2012 Mar 13;7(3):e33470. doi: 10.1371/journal.pone.0033470 (PMC3302775; doi:10.1371/journal.pone.0033470)

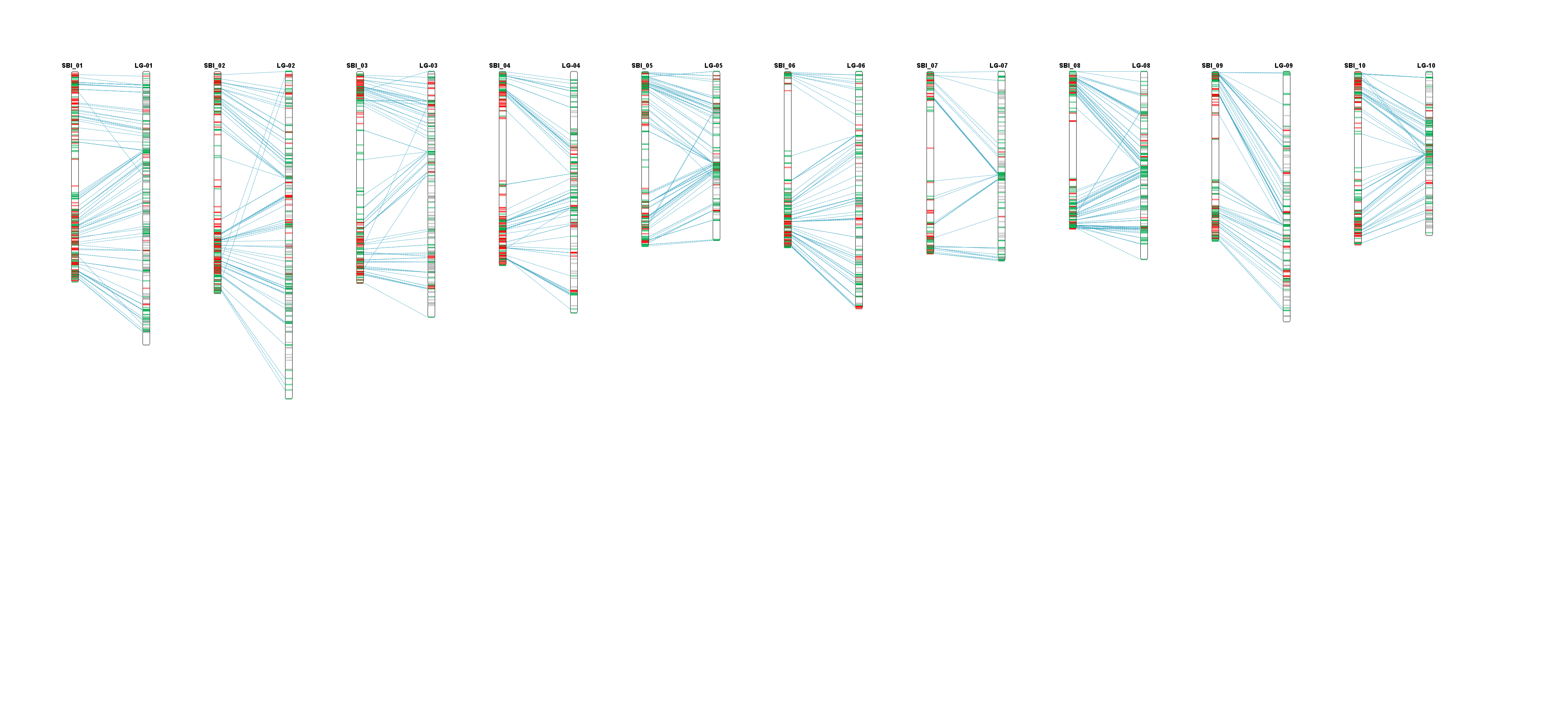

Supplement: Figure S1 — Collinearity of physical and genetic maps. This figure illustrates the genome coverage of the physical (SBI) and genetic (LG) maps of sorghum with the DArT genotyping tool. The genetic map (CIRAD Map mentioned in Mace [19] et al. 2009) includes 507 DArTs, 180 SSRs and 52 RFLPs. The physical map includes 1346 non-redundant DArTs (1412 loci), with 436 in common with the genetic map and 138 SSRs. Ninety-eight percent of the markers are collinear. PstI-BanII DArT markers are indicated in green, MITE DArT markers are indicated in red and SSR and RFLP markers are indicated in grey. (TIF) [file pone.0033470.s001.tif]

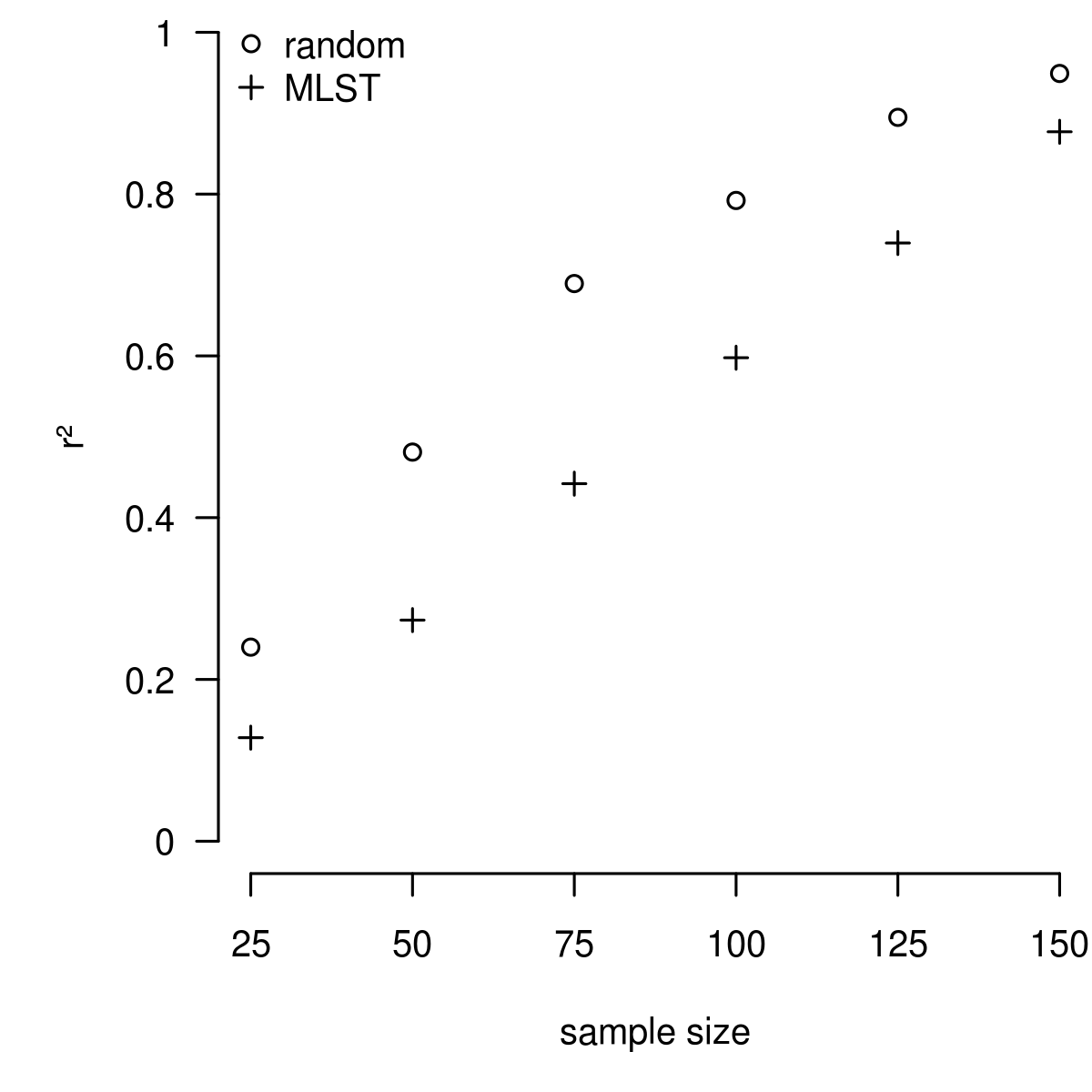

Supplement: Figure S2 — Correlations between linkage disequilibrium estimates obtained either with the Random or the MLST sampling strategies and the whole Core Sample. The increase in correlations with sample size was much faster with random sampling than with the MLST strategy. These observations probably come from the fact that in the random sampling strategy, the redundancy available in the whole CS was maintained in the sub-samples and the groups that are the most represented in the CS contributed more than those that are under-represented, leading to the same biases as those observed with the whole CS. (TIFF) [file pone.0033470.s002.tif]

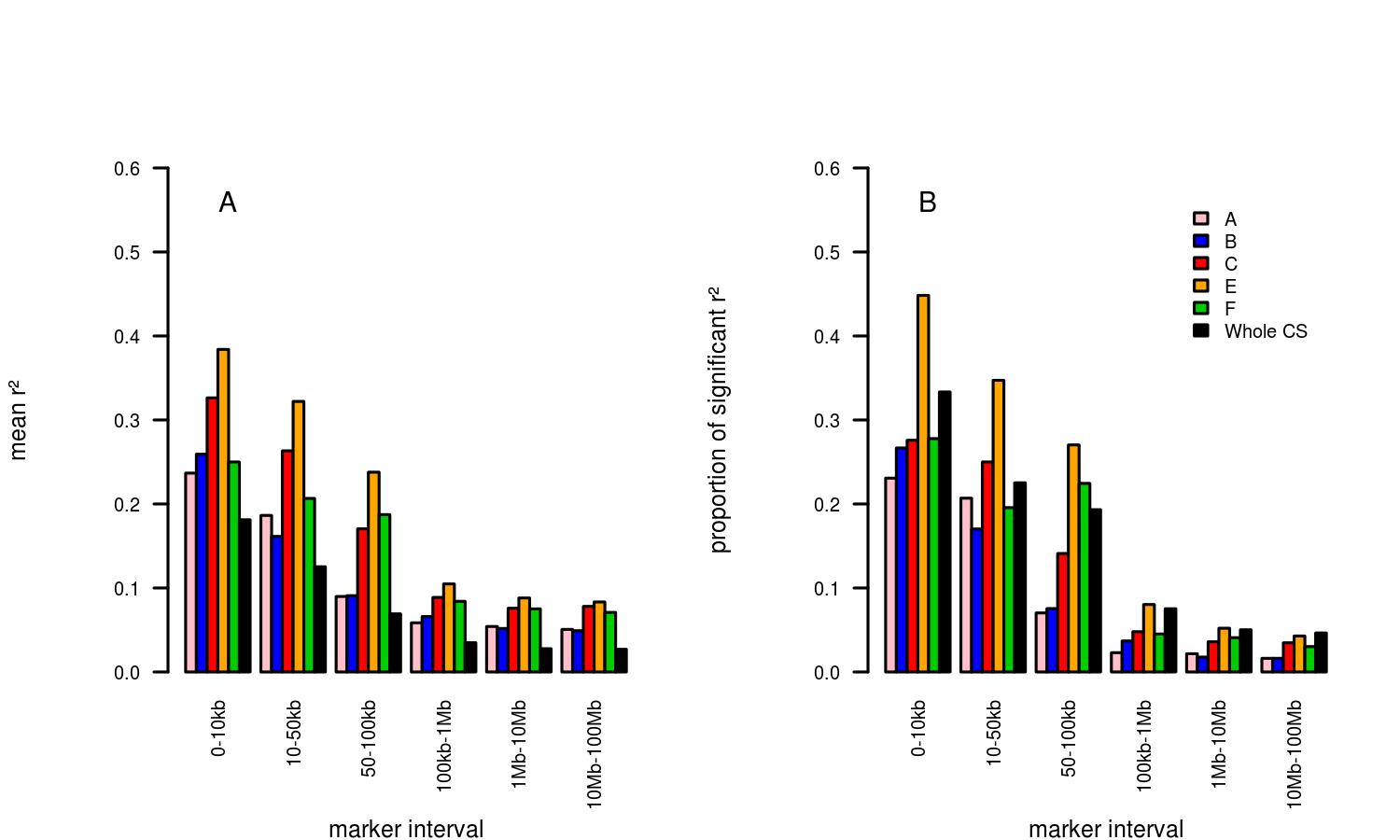

Supplement: Figure S3 — Evolution of linkage disequilibrium in the different genetic groups. Mean r2 (A) and the proportion of significant pairwise r2 (i.e. greater than P95) (B) were computed for the different genetic groups. Mean r2 were quite different between the genetic groups. Percentages of significant values were also variable between groups, with groups A and B, which were the most diverse, showing lower LD (r2 = 0.1 at 100 kb and less than 10% of marker pairs harbouring significant LD) and group E, which was the least diverse and the most recent, showing higher LD (r2>0.2 at 100 kb and more than 25% of marker pairs harbouring significant LD). (TIFF) [file pone.0033470.s003.tif]
